# Supplementary material for: Are carbon emissions trading and green financial instruments synergistic? -Comprehensive quantitative research based on content analysis
Source: PLoS One. 2024 Mar 7;19(3):e0298601. doi: 10.1371/journal.pone.0298601 (PMC10919601; doi:10.1371/journal.pone.0298601)
Supplement: S1 Data — (ZIP) [file pone.0298601.s001.zip › Data/地级市Z_F工作报告.zip!/╜¡╬≈/╛┼╜¡╩╨/└┤╫╘╣1⁄2╓┌║┼í╛┬φ┐╦╩2╛▌═°í┐_2011.docx]

各位代表：

现在，我代表市人民政府，向大会报告工作，请予审议，并请市政协各位委员和列席会议的同志提出意见。

一、2010年及“十一五”时期经济社会发展主要情况

2010年是极不平凡的一年。在省委、省政府和市委的坚强领导下，全市上下以科学发展观为指导，深入推进赶超发展战略，一手抓发展，一手托民生，夺取了防汛抗洪的重大胜利，完成了年初确定的主要目标任务。全年实现生产总值1032亿元，比上年增长14.3%；财政总收入116.7亿元，净增30.5亿元，增长35.4%；全社会固定资产投资900.9亿元，增长36.7%；规模以上工业增加值360.6亿元，增长22.4%；外贸出口12.1亿美元，增长162.3%；社会消费品零售总额284.1亿元，增长19.5%；居民消费价格指数上涨3.2%；城镇居民人均可支配收入15764元，增长11%，农村居民人均纯收入5588元，增长16%，在岗职工年平均工资24252元，增长15.2%。2010年各项目标任务的实现，显示出科学发展、奋起赶超的强劲势头，标志着“十一五”目标任务的圆满完成，为三年翻番、五年进位奠定了基础。

——经济总量不断扩大，综合实力不断提升。五年来，全市经济总量五年翻一番，财政总收入、社会消费品零售总额、金融机构存贷款余额四年翻一番，全社会固定资产投资、工业增加值三年翻一番，出口总额两年翻一番。生产总值年均增长13.7%，人均GDP超过3000美元；财政总收入年均增长24%，五年增长1.93倍；全社会固定资产投资年均增长36.1%，五年增长3.67倍；社会消费品零售总额年均增长18.6%，五年增长1.34倍；规模以上工业增加值年均增长24.3%，五年增长2.85倍；出口总额年均增长59.4%，五年增长9.3倍。

——经济结构不断优化，发展质量不断提高。五年来，三次产业比由16.8∶50.1∶33.1调整为9.5∶56.2∶34.3，工业主导地位更加凸显。财政总收入占GDP的比重由8.2%提高到11.3%，税收占财政总收入的比重由80.6%提高到88.1%，县级财政总收入占全市的比重由67%提高到80%。非公有制经济加快发展，民营企业达1.8万户，上缴税收占财政总收入“半壁江山”。科技对经济增长的贡献率提高8个百分点。旅游经济主要指标总量连续五年居全省首位。生态建设扎实推进，森林覆盖率提高到54.7%，城镇生活污水集中处理率达95.6%，城乡生活垃圾无害化处理率达66%，节能减排任务全面完成。

——“三化”进程不断加快，城乡面貌不断改观。五年来，特别是市委九届七次全会以来，按照“两区互动，强工兴城”的工作重点，以新型工业化支撑新型城镇化，以适度超前的城镇化带动工业化，找到了一条九江加快赶超的新路子。规模以上工业企业达904户，净增409户。主营业务收入过亿元企业319户，净增232户，过10亿元企业17户，九江石化突破200亿元，九江钢厂突破100亿元。星火有机硅扩改、九江电厂“上大压小”、九江石化、九江钢厂、旭阳雷迪、江铜铅锌冶炼等一批重大产业项目取得重大突破，九江经济技术开发区升格为国家级开发区。城镇化率提高到46.5%，尤其是近两年来，投入145.8亿元开工和建成一大批重大项目，大力推进中心城区从“两湖”时代走向八里湖时代，“四县一山”与中心城区功能对接步伐加快，“大九江”格局已见雏形。县城建设亮点纷呈，集镇功能逐步完善。全面落实强农惠农政策，取消了农业税，粮、棉、油等大宗农产品产量稳步增长。建立省级以上无公害、标准化农业基地92个，培育农产品生产基地140个、规模以上农业企业302户、农民专业合作社1350家。完成6640个自然村新农村建设和农村清洁工程试点，惠及110万农民，新农村建设连续四年保持全省领先。改造农村危房4066户，移民扶贫搬迁深山区群众3.5万人，357个贫困村整村推进成效显著。

——基础设施不断完善，发展后劲不断增强。五年来，完成交通设施投资近300亿元，综合交通现代化进程加快。铜九铁路、昌九城际铁路和武吉、彭湖、九瑞高速建成通车，铁路和高速公路运营里程分别增加到350公里和374公里，高速铁路实现历史性突破。改造316国道、柯龙线修水段等国（省）道277.5公里。新增3000吨级以上泊位12个，新增吞吐能力1280万吨，城西港区30万标箱集装箱码头建成投运。长江二桥开工建设，九江机场顺利复航。九昌樟成品油管线基本建成，“西气东输”、“川气东送”九江段建成投运。累计完成电网投资34亿元，统调电力装机达218万千瓦，都昌矶山湖、星子大岭、庐山区长岭等风电项目并网发电，彭泽核电前期工作进展顺利。改造农村公路5400公里，渡改桥73座，完成510座小（二）型以上病险水库除险加固，改造一批中型排涝泵站。基本实现村村通油（水泥）路、通广播电视、通电话、通宽带。农业生产生活条件进一步改善。

——改革创新不断深入，对外开放不断扩大。五年来，国企改革取得重大突破，126户国有工业企业改制全面完成，安置职工8万多人，累计发放、补缴“一补三险”20多亿元。非工口六大系统98户国企改革按期完成，农垦系统14户国企改革平稳推进。集体林权制度改革经验在全国推广。鄱阳湖采砂统一管理成效显著。新一轮政府机构改革和财政综合改革全面完成。医药卫生体制和户籍制度改革全面启动。完善国资监管和投融资体制，组建一批投融资平台，引进并做大一批金融机构。对外开放进一步扩大，新批外资项目708个，实际利用外资23.6亿美元，引进内资1171亿元，出口总额达23.3亿美元，外贸依存度由3%上升到8%。新增6对国际友好城市。

——人民生活不断改善，社会事业不断发展。五年来，城镇居民人均可支配收入、农村居民人均纯收入、在岗职工年平均工资、居民储蓄存款年均分别增长12.6%、11.3%、14.7%、18.7%。民生工程扎实推进，实现了城乡困难群众最低生活保障、城乡困难群众大病医疗救助、城乡义务教育免学杂费和贫困生资助政策、城乡基本医疗保险制度“四个全覆盖”。历年“两会”上承诺的313件惠民实事基本完成。投资15.4亿元，建设29625套保障性住房，改造棚户区115万平方米，发放租赁补贴3631万元，惠及6万户低收入住房困难家庭。解决了2万余户房产发证历史遗留问题。义务教育全面普及，学前教育、特殊教育、职业教育不断加强，高等教育不断提升。科技人才事业明显进步，共获省级以上科技进步奖15项、专利授权692项，引进外国专家200余人。社区卫生服务网络基本建成，甲型H1N1流感、手足口病、血吸虫病等疾病得到有效防控。文化事业加快发展，全民健身广泛开展，竞技体育屡创佳绩。人口自然增长率下降0.3‰，出生人口性别比过高问题得到有效遏制。“五五”普法全面完成。安全生产、社会治安综合治理不断加强，公众安全感不断提升。

2010年是实施“十一五”规划的最后一年，围绕全面完成既定的目标任务，主要抓了以下六个方面工作：

（一）全面加大强工力度，工业经济实现新扩张。围绕三年决战工业2000亿的决策部署，进一步营造强工氛围。市财政拿出1000万元奖励贡献突出的工业企业及园区，1000万元支持企业技改创新。健全重大工业项目推进机制，推行“一名领导、一套班子、一事一议”的服务机制和“一周一督查，一月一调度，一季一通报，半年一考核，年终总评比”的督查考核机制。同时，大力支持和激励县区提升工业发展水平。围绕做大工业总量，加快推进工业项目。工业固定资产投资618亿元，增长42.9%，占全社会固定资产投资的68.6%。巨石17万吨玻纤、旭阳雷迪二期、龙达11万吨粘胶纤维、中粮30万吨植物油、赛得利9万吨粘胶等一批重大项目投产。攀森25万吨多金属镍粒、亚东水泥600万吨扩能、旭阳雷迪三期等一批重大项目开工建设。规模以上工业企业主营业务收入迈上1500亿元新台阶，增长47.6%，利税总额163.9亿元，增长39.9%，工业经济总量居全省第二。围绕提升平台承载力，大力做强工业园区。全市建设标准厂房47万平方米、公租房106万平方米，园区开发面积达82.4平方公里。庐山区获批国家玻璃纤维及复合材料高新技术产业化基地，蓝星星火获批国家级有机硅企业技术中心。园区主营业务收入突破1000亿元，九江经济技术开发区、湖口工业园突破200亿元，永修云山经济开发区、瑞昌工业园突破100亿元。

（二）着力保持兴城势头，城市建设迈出新步伐。按照新一轮城市总体规划，加快构建“大九江”格局。完成城市总体规划和八里湖新区控规修编，中心城区控规覆盖率达98%。加强“大九江”范围内交通对接，九码快速通道、八里湖大道建成通车，105国道环庐山段拓宽改造等项目加快推进，绕城高速、蔡马高速启动前期工作，“四县一山”与中心城区相向融合步伐加快。按照“三年决战200亿”的目标，大力推进中心城区建设。全年续建、新建、启动项目118个，完成投资过百亿元。体育中心、八里湖公园、八里湖东路、新区学校和新区医院主体工程等一批项目完工，八里湖大桥、规划展示馆等项目加快推进，新区框架全面拉开，路网基本成型，功能逐步完善。老城区濂溪大道、十里河整治、火车站改造、龙开故道公园等项目全面竣工，滨江路、长虹北路、浔阳东路、长江大道、九瑞大道等改造升级，浔阳东路地下人防设施、大中路改造等一批工程基本建成。滨江生态公园改造、庐山区整区改造、浔阳区分片改造全面启动。城区新增道路里程64公里、路面171万平方米，路网沥青改造总面积102万平方米。加大城区土地、资产经营和规费收缴力度，有效保障了城建项目资金需求。按照“六城同创”的决策部署，不断提升城市管理水平。创建全国无障碍建设城市通过验收，实现全省双拥模范城市“六连冠”。中心城区十大农贸市场标准化改造、百条背街小巷整治全面完成。市容环境、交通秩序等专项整治力度进一步加大，“两声三乱”整治成果得到巩固，“文明祭扫”活动成效明显。城区违章建筑得到有效遏制，农民建房进一步规范。同时，各县（市、区）城市框架在拉大，功能在完善，精品在增多，品位在提升，共启动城建项目685个，总投资377.5亿元，开工项目634个，竣工328个，完成投资176.4亿元。

（三）进一步扩大投资规模，项目建设取得新成效。通过向上争资，推进一批重大基础设施项目。全市28个项目列入省重点工程，完成投资152.4亿元。京九线、武九线电气化改造完成，武九客专、九景衢铁路等项目通过核准，池九城际铁路、九岳铁路、咸宜井铁路等项目取得实质性进展。通过招商引资，推进一批重大产业项目。引进市外3000万元以上项目447个，其中亿元以上172个，10亿元以上18个。实际利用外资6.7亿美元，增长27.8%。理文造纸、铨讯电子、赛龙手机等一批产业项目落户。实际利用外资、引进内资、外贸出口增幅和开放型经济综合考评均获全省第一。通过加大投入，推进一批民生社会事业项目。市特教学校康复楼、科技中专新校区、浔阳区全民健身中心、自来水一户一表改造和县区老年公寓等项目加快推进，市新客运汽车总站投入运营，中医院南院竣工，市农产品质量安全监测中心、儿童福利院、广电演播中心、妇幼保健院住院大楼等交付使用。通过强化保障，加快项目推进步伐。扎实开展项目建设大会战，纳入“五线调度”的552个项目快速推进。加强政银企合作，市本级融资平台新增融资38.5亿元。5家小额贷款公司提供贷款5亿元，37家市县担保机构提供贷款担保20.2亿元，有效保障了资金需求。

（四）不断夯实赶超基础，统筹发展取得新进步。扎实推进“三农”工作。“十百千”现代农业示范工程顺利推进，建设万亩示范区11个、千亩示范片107个，新扩城郊蔬菜基地4000亩。新增规模以上农业加工企业51户，新增3000万元以上农业项目114个，其中亿元以上48个，新增农民专业合作社285家，带动农户6.5万户。新增省级以上农产品品牌8个、无公害有机绿色农产品86个。1132个自然村“三清七改五普及”全面完成，3.4万户农民用上自来水，沼气池保有量达22万户。农业产业化和农村清洁工程进入全省先进行列。在全省率先启动农村信息化体系建设试点。发放农业各项补贴3.4亿元，补贴汽车、摩托车、家电下乡资金1.47亿元。全面完成基层农技推广体系改革，建成综合站181个，全国现场会在我市召开。农村土地流转试点进展顺利，林权制度配套改革稳步推进，种子、畜牧、水产管理体制改革基本完成。扎实开展扶贫、移民工作，完成投入超3亿元。大力发展县域经济。进一步加大支持力度，完善考核激励机制，扩大审批事项下放范围，以多种形式帮扶解决发展难题，引导争先进位，促进竞相发展。实现了县县增收超亿元、县县总量过三亿、两个县区超十亿、四县增幅超五成、五县增幅进全省前十。支持共青城超常规发展，共青城设市并被列为全省统筹城乡经济社会发展一体化试点区。开展“示范镇”和“扩权强镇”试点工作，一批重点乡镇加快发展。积极建设生态文明。造林绿化147.6万亩，治理水土流失200万亩。新增国家级生态村1个、省级生态乡镇17个、省级生态村15个。加强重点耗能企业节能监察，实行工业企业差别电价，开展公共机关节能活动。鹤问湖污水处理厂建成投运。积极开展环保专项行动，强化污染减排，一批企业退城进园，扩能提升。发展绿色生态旅游，庐山南北山门及换乘中心建成投运，中信西海旅游综合开发、东林大佛景区等一批项目开工建设。全年接待游客1977.9万人次，实现旅游总收入142.9亿元。

（五）扎实推进社会建设，人民生活有了新改善。民生工程全面完成。整合民生资金32.8亿元，100件惠民实事基本完成。新增城镇就业8.5万人，“4050”人员再就业8380人，“零就业”家庭就业实现动态清零。发放创业小额贷款6亿元。就业培训5.1万人，新增转移农村劳动力就业7.1万人，城镇登记失业率控制在4％以内。城镇职工养老保险参保率达75％，养老保险基金征缴总量突破13亿元。新农合参合农民达342万人，年人均筹资提高到150元，补偿封顶线提高到5万元。中心城区低保标准月人均提高到300元，农村低保标准月人均提高到120元。城镇企业退休人员养老金月人均增加150元。农村五保户集中供养率达85％。完善困难群众医疗救助制度，建立四级定点医疗救助体系。城镇居民基本医疗保险参保人数达85.5万人。“光明·微笑”工程转入常态管理。开工建设公租房50万平方米。新建廉租房28.3万平方米，为1.77万户发放租赁补贴，为700户发放经济适用房货币补贴。扎实开展市场整治，发展环境进一步优化。加强价格监管，粮食、猪肉、蔬菜等价格涨幅均低于全省平均水平。各项事业全面发展。深入推进科技创新“六个一”工程，实施科技开发项目43个，引进推广新技术、新产品100项。新建省级重点实验室和工程技术中心2家。强化技能人才队伍建设，启动实施特别职业培训计划，培养中高级人才1万多人。农村中小学教育条件持续改善，师资队伍得到加强，城区学校网点布局逐步优化，市内各高校服务地方发展作用进一步显现。九江画院、市博物馆、乡镇文化站等文化基础设施项目加快推进，“农家书屋”建设任务超额完成。扎实开展全民健身运动，农村体育基础设施进一步完善。人口计划生育工作全面提升，第六次人口普查进展顺利。审计、海关、检验检疫、应急管理、外事侨务、气象、水文、民族宗教、无线电管理、档案、保密、地方志等工作进一步加强。国防教育、征兵、民兵预备役、人民防空、防震减灾、国家安全等工作取得新成绩。工会、共青团、妇女儿童、老龄、残疾人、红十字和慈善等事业实现新发展。社会保持和谐稳定。全面落实安全生产责任制，深入开展隐患排查整改，大力推进安全标准化建设，安全生产形势进一步好转，各类伤亡事故和死亡人数分别下降4.1%、16.3%。完善信访调处机制，拓展信访渠道，群众合法诉求得到有效解决。扎实推进社会治安防控体系建设，不断强化公共安全监管，“两项”整治等专项行动成效明显，“天网”工程一期建设全面完成，在全省公众安全感测评中名列第三。

（六）努力打造高效政府，自身建设得到新加强。围绕“核心党委、高效政府”的格局，切实把市委的决策部署化为政府的目标任务和实际工作，积极作为，高效执行，奋力赶超。突出强工兴城重点，着力破解改革、融资、土地运作等难题。推进法治政府建设，主动接受市人大及其常委会的法律监督和工作监督，自觉接受市政协的民主监督和社会舆论监督，充分听取各民主党派、工商联的意见建议，加强与工会、共青团、妇联等群众团体联系。办理市人大代表建议256件、市政协提案460件，研究办理市人大常委会审议意见建议67件，办结率均为100%。围绕“一手抓发展、一手托民生”的理念，将抓发展与托民生、开拓新城与提升老城、推进兴城与推进“三农”、全面强工与发展三产、引进外资与激活民资、做强市本级与做强县域经济等方面统筹考量，协调推进。通过县区、战线办公会，及时研商解决难题，做到“有请必示、有求必应、有困必帮、有急必救”。以民声直通车为平台，创新完善“12345，有事找政府”的服务模式，受理群众诉求6671件，办结率99.9%。围绕“对党负责，为民服务”的宗旨，完善政府运行机制，通过联动省市县考核量化目标，通过梳理“六张作业单”细化任务，通过推行“三线工作法”优化运行，通过调动多方积极性强化合力，打造合心合力合成的高效团队。积极开展“创业服务年”活动，扎实推进民主评议政风行风和基层站所，严肃查处破坏发展环境和损害群众利益的案件，建立重点案件督办、回访制，效能投诉办结率达99%以上，“创业服务年”活动考评名列全省第二。深入推进廉政建设和反腐败工作，加强对工程建设、土地、民生等领域突出问题的专项治理，维护了群众的合法利益。进一步规范公务支出，公务接待、公务用车、因公出国（境）费用实现“负增长”。

各位代表，成绩来之不易，经验更须坚持。回顾五年来的工作，我们经受了诸多严峻的考验和挑战，全面取得了抗震救灾、抗冰救灾、抗洪救灾的重大胜利，有效应对了国际金融危机的严重冲击，努力保持了经济社会的平稳较快发展。特别是近两年来，我们在市委的坚强领导下，紧紧依靠全市人民，通过强工使经济提速、实力增强，通过兴城使面貌变样、活力增强，通过项目建设使结构优化、后劲增强，通过统筹协调使民生改善、基础增强，通过改革创新使发展加快、动力增强。

我们深刻地体会到：两区互动、强工兴城，是赶超发展的主攻重点；狠抓项目、扩大投资，是加快赶超的主导力量；以人为本、改善民生，是赶超发展的终极目标；改革创新、务实操作，是推动赶超的强大动力；共识共为、合力合成，是实现赶超的根本保障。

我们深切地感受到：成绩的取得，是省委、省政府和市委坚强领导的结果，是市人大、市政协监督支持的结果，是全市人民和各界人士团结奋斗的结果。在此，我代表市人民政府，向辛勤劳动在各个领域的全市人民，向大力支持政府工作的各位人大代表、政协委员，向离退休老同志，向各民主党派、工商联、各人民团体和社会各界人士，向驻浔部队和武警官兵，向中央和省驻浔各单位，向所有关心支持九江发展的朋友们、各位纳税人，表示衷心感谢并致以崇高敬意！

我们清醒地认识到：经济总量不大、发展不足的市情尚未根本改变，赶超进位的任务仍然艰巨；工业化、城镇化水平不高的局面尚未根本改变，强工兴城的任务仍然艰巨；群众收入水平偏低、部分居民生活还比较困难的现状尚未根本改变，提高收入和改善民生的任务仍然艰巨；影响和谐稳定的因素依然存在，社会建设和管理面临的任务仍然艰巨；发展环境有待进一步优化，依法行政水平有待进一步提高，政府自身建设有待进一步加强。对此，我们将采取更加有力的措施，积极应对，努力解决。

二、“十二五”时期经济社会发展总体安排

“十二五”时期，虽然国际形势复杂多变，但我国发展基本面将总体向好，我省将以鄱阳湖生态经济区建设为龙头，快速推进新型工业化和新型城镇化。对九江而言，这既是难得的战略机遇期，也是既定的赶超决战期。按照市委九届十二次全会精神，全市经济社会发展的指导思想是：高举中国特色社会主义伟大旗帜，以邓小平理论和“三个代表”重要思想为指导，深入贯彻落实科学发展观，以加快转变发展方式为主线，以鄱阳湖生态经济区建设为龙头，坚持赶超发展战略不动摇，继续强工兴城，促进统筹发展，加速推进新型工业化、新型城镇化和农业农村现代化，着力提高人民群众幸福指数，努力实现赶超进位。

“十二五”全市经济社会发展总体目标是：九江争得应有地位，人民过上小康生活。具体目标是：生产总值年均增长13%以上，实现翻番；财政总收入年均增长20%以上，突破300亿元；全社会固定资产投资年均增长25%以上；工业主营业务收入年均增长25%以上，突破5000亿元；工业化率年均提高1个百分点；城镇化率年均提高1.5个百分点；城镇居民人均可支配收入、农村居民人均纯收入和在岗职工年平均工资年均增长10%以上；完成节能减排任务。

为实现上述目标，我们将着力抓好六个方面的工作：

（一）坚定不移推进鄱阳湖生态经济区建设，着力构建对江西崛起具有重要贡献的发展极。紧紧抓住鄱阳湖生态经济区建设上升为国家战略的重大机遇，充分发挥九江区位和生态优势，在发展中转变增长方式，在转变中优化经济结构，在做大总量中提高发展质量；创新机制，先行先试，加快形成有利于节约能源资源和保护生态环境的产业结构、增长方式和消费模式，促进人与自然、物质文明与生态文明协调发展，努力实现在鄱阳湖生态经济区中率先崛起。

（二）坚定不移推进强工兴城，着力加快新型工业化和新型城镇化进程。突出搭平台，攻招商，强企业，促集群，加大强工力度。围绕“三十而立，保五争六”的目标，培植主营业务收入100∽500亿元的产业10个以上，主营业务收入过100亿元的企业10户以上，新增主营业务收入50亿元的企业10户以上；规模以上工业主营业务收入突破5000亿元，力争6000亿元。以沿长江、沿昌九为主平台，做大骨干企业，做强主导产业，发展战略性新兴产业，重点培育石化、有色冶金、纺织服装、汽车、船舶、电子信息、新能源、非金属新材料、节能电器、绿色食品等十大产业集群，形成一批在全省乃至全国有重要影响的优势产业、特色产业、骨干企业。突出拉框架，建新城，改老城，强县城，扩集镇，保持兴城势头。积极融入环鄱阳湖城市群和沿京九城镇密集带，完善市域城镇体系，以快速通道为纽带，构建1400平方公里的“大九江”城市发展格局；以基础性、功能性项目为支撑，基本完成八里湖新区建设；以庐山区整体改造和浔阳区分片改造为重点，完善老城区功能；加快县城和中心集镇发展，促进城乡经济社会一体化；加快提升城市经营和管理水平，着力打造宜居宜业城市。

（三）坚定不移把握统筹兼顾的根本方法，着力促进协调发展。坚持“三农”基础地位不动摇，以工业化转移农民、以城镇化带动农村、以产业化提升农业。新增规模以上农业龙头企业100户以上。粮食种植面积稳定在400万亩左右，粮食总产稳定在150万吨以上。完成新农村建设点5000个，加强农村基础设施建设，农村垃圾无害化处理率达80%以上，全面改善农村生产、生活环境。加快扩权强县、兴乡强镇步伐，健全激励机制，促进县域经济扬优发展。力争县县财政总收入过10亿元，一半以上过20亿元，1∽2个县进入全省十强，多数县进入全省前50位。整合旅游资源，推动庐山旅游整体上市，打造城区旅游休闲、庐山西海运动养生、共青城旅游商贸、庐山国学文化等旅游板块，构建以庐山为龙头的“大旅游”格局，旅游经济主要指标年均增长10%以上。大力发展现代物流、金融保险、信息咨询和科技服务等生产性服务业，积极培育新型消费业态。

（四）坚定不移实施重大项目带动战略，着力增强经济社会发展后劲。交通方面：建设九景衢铁路、武九客专、池九城际，实施九江火车站改扩建，开工建设合九客专、咸宜井铁路，启动九岳铁路前期工作；改造九江机场，建成永武高速、长江二桥、绕城高速、蔡马高速、昌九高速扩建等工程，实现省际、市际出口及市县通道标准均达二级以上；做大城西、城东港区，建设九江港煤炭储配中心码头、城西港铁路专线，推进中石化散装码头改造、彭泽核电配套码头等一批专用码头建设，构建多式联运的综合交通体系。能源方面：基本建成彭泽核电一期，争取启动二期项目前期工作；建成武宁下坊、修水三都水电站；建成都昌老爷庙等一批风电项目；建成马回岭500KV变电站和石钟山变电站至景德镇500KV输变工程，新建、扩建220KV变电站14座；加快推进天然气供气管网建设，力争实现所有县城及重点工业园区天然气管网全覆盖。水利设施方面：积极推进鄱阳湖水利枢纽工程建设，启动“引柘入城”工程规划论证，推动城市防洪排涝、长江堤护岸、病险水库除险加固等重点水利工程。信息网络方面：加速通讯、有线电视、互联网“三网”融合，建设全市统一的信息平台，积极打造“数字九江”。

（五）坚定不移推进改革开放，着力增强发展活力和动力。深化行政管理体制、国有企业、要素市场、投融资体制等改革，畅通民间投资渠道，大力发展非公有制经济。实施财政、户籍、收入分配等综合性改革，推进教育、卫生、科技、文化等领域改革，促进社会公共服务均等化。实施人才强市战略，加快科技创新平台建设，促进科技成果转化。继续实施“大开放”战略，围绕十大产业集群开展产业招商，主动承接产业转移，鼓励企业开拓国内、国际市场，提升开放型经济发展水平。充分发挥国家一类水运口岸和出口加工区的优势，努力做大对外贸易规模。

（六）坚定不移保障和改善民生，着力提高人民群众幸福指数。坚持富民为先、民生为重，开发就业岗位，开展职业培训，加大就业创业帮扶指导力度，推动能人创企业、百姓创家业、干部创事业，促进群众增收与经济发展基本同步。以基本养老、基本医疗、最低生活保障为重点，不断提标扩面，实现人人享有基本生活保障。加快医药卫生体制改革，巩固新农合成果，促进基本公共卫生服务均等化。加快构建多层次住房供应和保障体系，大力实施“安居”工程，中心城区建设500万平方米、10万套公租房，致力于让新市民住得下、留得住，让老市民住得新、住得好，让困难群众有房住，让企业职工能安居，让城市家园更美好，努力把九江打造成为“只要在九江就业创业，就能在九江安居乐业”的和谐幸福之城。积极发展社会福利事业，提高弱势群体基本生活水平。促进公共教育和文化资源向农村、贫困地区倾斜，丰富群众精神文化生活。深入开展全民健身运动，提高群众健康水平。完善突发公共事件应急管理体系建设，加强社会治安综合治理，维护社会和谐稳定。在此基础上，我们将依据《十二五规划纲要》，专门编制《民生发展专项规划》，进一步将民生改善目标化、具体化、责任化，促进民生全面、协调、可持续改善。

各位代表，九江正处于政通人和、快速发展的阶段。站在新的起点上，我们将始终依靠省委、省政府和市委的正确领导，始终依靠市四套班子的共识共为，始终依靠全市人民的共同努力，坚持好思路，保持好势头，努力实现九江争得应有地位、人民过上小康生活！

三、2011年工作任务

2011年是“十二五”规划的首战之年，是“三年翻番”的决战之年，也是“五年进位”的关键之年。今年的政府工作，将在省委、省政府和市委的坚强领导下，认真贯彻落实科学发展观，坚持赶超发展战略不动摇，通过实施两区互动、项目推动、统筹联动，一手抓发展促赶超，一手托民生促和谐，实现三年翻番，为五年进位和“十二五”目标的完成奠定坚实基础。

全市经济社会发展的主要预期目标是：生产总值增长13%，力争15%以上；财政总收入增长20%，力争30%以上；全社会固定资产投资增长25%，力争30%以上；社会消费品零售总额增长18%；城镇居民人均可支配收入、农村居民人均纯收入和在岗职工年平均工资增长10%以上；出口总额增长20%以上；居民消费价格指数控制在4%以内；城镇登记失业率控制在4.5％以内；人口自然增长率控制在8‰以内；完成省政府下达的节能减排任务。

为实现上述目标，我们将继续竭尽全力抓发展，不遗余力托民生，推动“发展加速、基础加强、民生加力”，重点做好七个方面的工作：

（一）加速做大工业总量，提前一年实现2000亿目标

推进企业做大。以现有骨干企业为依托，提升龙头企业的带动力，加快推进九江石化、九江钢厂、亚东水泥、星火有机硅等重大项目扩能升级；以重大产业项目为依托，提升重点产业的牵引力，开工建设彭泽核电一期，加快推进旭阳雷迪三期、江铜铅锌冶炼、理文造纸、攀森多金属镍粒、龙达化纤二期、巨石玻纤二期等一批项目；以央企省企对接为依托，支持企业靠大联强，提升核心竞争力，加快推进新钢与萍钢的战略性合作、昌铃汽车的战略性布局以及金源化纤的资产重组。力争规模以上工业增加值增长20％，主营业务收入增长30%，利税总额增长20%，新增1户主营业务收入过100亿元、5户过30亿元的企业。

促进产业集聚。坚持规划引领，加快完善全市产业发展规划，引导同类企业、关联企业抱团扎堆，进一步优化工业产业布局；实施龙头带动，发挥龙头企业在产业集聚中的辐射、吸引和带动作用，注重引进上下游配套项目，延长产业链条，扩大产业规模；创新工作机制，建立“一个产业、一名领导、一套班子、一个方案”的产业发展推进机制，实行重点产业项目、骨干企业挂点帮扶。建立十大产业集群分类统计体系，完善调度、督查、考核办法。加大产业集聚奖励力度，对获得国家级、省级产业基地的，市财政给予一次性奖励。力争十大产业集群主营业务收入达到1700亿元。

完善园区配套。加快水、电、路、通讯等基础设施建设，进一步提升基础性配套水平；建设一批园区物流中心、企业研发中心和公共创新平台，进一步提升生产性配套水平；推进公租房建设，加快发展园区周边商贸零售、餐饮配送、休闲娱乐等服务业，进一步提升生活性配套水平。优化园区用工环境，加大企业用工、培训补贴力度，推进园区用工对接，有效缓解企业用工难题。加大园区闲置土地清理处置力度，提高单位面积的投资强度、主营业务收入、上交税金和就业贡献率。园区主营业务收入增长40%以上，新增2家过百亿园区。

（二）加速推进兴城步伐，提前一年实现200亿目标

坚持规划引领，构建“大九江”格局。完成“大九江”城镇群空间战略发展规划、综合交通体系规划，完善中心城区控制性详规修编，构建以中心城区、“四县一山”、重点集镇为支撑的“大九江”规划体系。积极推动交通对接，加快105国道环庐山段改造等骨干道路建设，启动轻轨交通规划研究，预留“四县一山”与中心城区的通道，努力在构建“大九江”20分钟快速交通体系上取得新进展。积极推动功能对接，加快推进“四县一山”与中心城区同步发展、融为一体。

强化精品意识，加快项目建设。按照“续建一批、开工一批、启动一批、规划一批”的部署，市本级安排投资100亿元以上，在中心城区实施120个重点项目。加快推进八里湖跨湖大桥、蛟滩水利综合整治工程、规划展示馆、博物馆、新区路网等项目建设，开工建设市民服务中心、八里湖公园二期、艺术中心、新区接待中心、新区消防站等公共设施，完善新区公共服务配套。加快推进滨江生态公园、滨江西大道改造步伐，开工建设长虹东大道、十里大道延伸线、浔阳西路二期等项目，启动浔阳路、三马路、老九湖公路、老九星公路改造。以庐山区“整区推进、整体提升”和浔阳区“分片推进、整体提升”为重点，加大老城区、城中村、危旧房改造力度。加快推进县城和重点集镇建设，不断提高新型城镇化水平。进一步健全施工质量保证体系和建设监管体系，大力整治和打击围标、串标行为，对项目建设实施全过程公开，打造阳光工程；实施全过程监理，打造优质工程；实施全过程监督，打造廉洁工程。

突出以人为本，加强城市管理。以“防堵治堵”为重点，加强交通设计和交通组织，加速开发利用城市地下空间，加快公共停车场建设，倡导文明出行，优先发展公共交通，大力缓解城市交通拥堵状况，着力打造畅通之城；以加强管理为重点，进一步完善城市管理体制，建立园林绿化管护、市政设施管养、环境卫生管控、小区物业管理的有效机制，巩固“两声三乱”和“文明祭扫”等专项整治成果，抓好市容环境、违法建设和城区砂石站等专项整治，着力打造文明之城；以开展创建为重点，深入推进“六城同创”，突出街道、社区和市民的主体作用，不断提升市民文明素质，力争成功创建省级生态园林城市和国家园林城市，着力打造生态之城。

发展第三产业，繁荣城市经济。稳定大宗消费，扩大传统消费，培育社区服务、养老保健等新兴消费热点，推进特色商业街区、星级宾馆、高档写字楼、美食街区等功能设施建设，提升综合服务能力。培育发展金融企业、商务服务、教育培训、现代物流、信息服务、专业市场等生产性服务业。加快21世纪家居中心、国际汽车城、华东建材市场升级改造等项目建设，支持有条件的县区培植区域性特色物流市场，推进共青城纺织服装工贸城、都昌珍珠交易市场、德安陆路物流港等市场建设。

（三）加速推进项目建设，进一步扩大固定资产投资规模

全力开展产业招商，提升招商引资水平。围绕“企业做大、产业集聚”，进一步解放思想，加强激励，加大产业招商力度，依托骨干企业引进上下游配套企业，不断壮大关联产业。重点面向港澳台，对接长珠闽，积极跟进央企和省属企业，大力开展招商推介，有效承接产业转移。确保实际利用外资增长10％以上，引进内资增长25％以上。

积极向上争资争项，改善基础设施条件。主动策应国家和省“十二五”规划，扎实做好国家投资项目的谋划、编报和争取工作，确保争资争项高于全省平均水平。开工建设武九客专、九景衢铁路和红光、浔东、姑塘、码头等输变电工程，加快推进长江二桥、永武高速、绕城高速、老爷庙风电、九江港煤炭储配中心等项目，配合做好鄱阳湖水利枢纽工程和九江电厂第2台机组等项目前期工作，推进南昌至湖口段二级航道整治工程。跟进池九城际铁路、咸宜井铁路、九岳铁路等项目，积极争取永武高速延伸线项目。加快推进九江港水运口岸扩大开放，推进铁路进港，逐步实现多式联运。

大力发展非公经济，拓宽投资融资渠道。进一步改善非公经济发展环境，鼓励全民创业，扩大民间投资，促进非公经济加快发展。积极搭建返乡创业平台，加大政策支持力度，掀起返乡创业新热潮。强化政银企合作，引导银行加大对项目的信贷支持。做大市城投、置地、国资等公司，加大资产经营力度，增设融资性担保公司、小额贷款公司，积极支持一批优质企业上市融资。

强化帮扶服务措施，加快项目建设进度。进一步强化安商富商理念，完善“企业之声”受理、办理机制，帮助企业破解项目审批、用地、用工和煤电油运保障等难题。健全项目建设责任体系，完善进度督查、现场考评、奖惩激励机制，落实重大项目联审联批制、涉政事务代理制和挂点帮扶制，切实提高招商项目履约率、资金到位率和项目开工率。

（四）加强“三农”工作，继续巩固农业基础地位

大力推进农田水利基本建设。以国家大兴水利为契机，配合做好第一次全国水利普查工作，完成688座小（二）型以上病险水库除险加固。积极实施农田水利规划，推进修水、都昌等小农水重点县建设。加快机电泵站更新改造，完善渠系配套设施，新增、恢复有效灌溉面积8.8万亩，改善灌溉面积17.4万亩。推进农业综合开发，建设高标准农田8万亩。

加快推进农业产业化进程。认真落实国家对粮食生产的各项扶持措施，促进粮棉油等大宗农产品稳产增产。加快农业产业示范区建设，推进加工园区、龙头企业、示范基地、合作组织“四位一体”发展。力争引进3000万元以上农业项目70个，实际进资20亿元以上。大力实施“5656”工程，实现规模以上农业加工企业主营业务收入300亿元，增长27%。推进“十百千”工程，万亩高产示范区基础设施建设完成70%以上，千亩示范片基础设施建设全面完成。加强无公害、绿色、有机农产品基地建设，培育做大农产品品牌。开展百强示范合作社建设，大力发展农民专业合作社，促进农民增收。

扎实推进新农村建设。坚持把小城镇建设作为统筹城乡发展的重要抓手，高标准规划建设乡镇农民集中居住示范点，加强集镇和大村庄的基础设施配套，让农民充分享受改革成果和现代文明。全面启动395个贫困村扶贫开发工作，完成深山区移民4161人。抓好1100个新农村建设点“三清七改五普及”和“三绿一处理”工作，推进3268个试点村、60个农村集镇的垃圾无害化处理。深入推进“万村千乡”市场工程建设，推进家电、汽车和摩托车下乡以及家电以旧换新等工作，促进农村消费结构升级。

统筹推进农村综合改革。巩固完善基层农技推广体系改革成果，实施农业科技入户工程，提高农业科技成果转化率和贡献率。积极探索土地流转新模式，推进适度规模经营，遏制土地抛荒。推进农垦体制改革和林权制度配套改革，加大金融对农业生产支持力度，扩大信贷担保试点范围，用财政资金引导龙头企业参与担保体系建设。

（五）加强鄱阳湖生态经济区建设，加快转变发展方式

抓好生态建设，推动绿色发展。高标准组织鄱阳湖生态经济区战略规划和生态产业、基础设施、环境保护等专项规划编制工作，启动指标体系、生态补偿、湿地保护等专题研究。加强鄱阳湖核心保护区和修河流域的生态保护，强化流域水质监测，保护好鄱阳湖“一湖清水”。进一步提升鄱阳湖采砂统一管理水平。强化城乡饮用水源保护，抓好县级污水处理设施完善配套和运行管理。加强城市空气、噪音、烟尘环境综合治理。

做强县域经济，推动协调发展。继续把县域经济作为加快赶超最具潜力、最具活力的板块来抓，进一步加大政策支持力度，管理上放权，机制上放活，扩大县域发展自主权。建立市县联动机制和县域协调机制，全力支持县域向上争资争项，协调推进县域基础设施共享、城乡协同发展、产业联合打造。完善县域经济发展激励机制，对进入全省年度考核“十强县”、“十快县”的县（市、区）实行奖励，并在建设用地指标、工业发展资金、产业基地扶助资金和市级支农资金等方面给予倾斜。积极引导各县依托特色产业、龙头企业，提高产业发展水平。继续支持共青城市在新的起点上加快发展。力争新增2个县（市、区）财政总收入过10亿元、3个过8亿元，县县过5亿元。

壮大文化旅游，推动特色发展。打响庐山旅游品牌，增强庐山核心景区的带动力，形成以庐山为龙头的大旅游格局。加快建设中信西海旅游综合开发、星子佛教文化游览区、九江旅游集散中心、共青城奥特莱斯、大中路西段历史文化街区等项目，完善庐山新城建设，提升景区档次和文化内涵。科学开发地热资源，以星子、庐山西海等景区为重点，大力发展以养生、休闲为主的温泉特色游。做优精品线路，加快实现山上与山下、景区与城区、城市与乡村、旅游与休闲的良性互动。加强旅游营销策划，扩大九江旅游专列的影响力，积极开拓境外旅游市场。力争全年接待旅游总人数增长15%，旅游总收入增长20%。

强化科技人才支撑，推动可持续发展。

结合发展需求，创新引才、用才机制，重点培养引进领军型、创新型、技能型人才。大力实施科技创新“六个一”工程，促进科技成果向现实生产力转化，力争科技成果转化项目增长30%。深化科技体制改革，促进科学研究与高等教育有机结合，引导和支持创新要素向企业集聚，加快建立以企业为主体、市场为导向、产学研相结合的技术创新体系。大力培育高新技术产业，引进壮大新能源、新材料、新型制造业等战略性新兴产业，力争高新技术产业产值、专利申请和授权增长30%。鼓励企业发展循环经济，提高资源利用效率。加强共生、伴生矿产资源综合开发，鼓励余热余压发电，力争工业单位综合能耗下降3%，工业固体废弃物综合利用率提高2%。

（六）加力改善民生，不断提升人民群众幸福指数

夯实民生基础。整合各类民生资金35亿元以上，围绕扩大就业与创业、社会保障、公共服务、保障性住房建设、改善人民群众基本生活条件等八个方面，给足力量，下足功夫。大力开展就业培训，新增城镇就业5.54万人，新增转移农村劳动力5.52万人。新增发放小额贷款2.9亿元。进一步扩大城镇职工养老保险、新型农村养老保险、失地农民养老保险覆盖面。城市、农村低保人均月补助标准分别提高到200元、85元，农村五保户集中、分散供养标准分别提高到2400元、1560元，城镇“三无”特困群众月人均供养标准提高到350元；城市、农村精简退职老职工月人均救济标准分别提高到245元、205元；继续对城区1万户低收入家庭发放价格补贴。新型农村合作医疗统筹基金使用率达到85%、一次性报账率达到90%。

办好惠民实事。在夯实民生基础的同时，实施以救困、救残、救病、救灾为重点的“温暖”工程；以加快蔬菜基地建设为重点的“菜篮子”工程；以加快公租房、廉租房建设和棚户区、农村危旧房改造为重点的“安居”工程；以提高通行能力、方便市民出行为重点的“城市畅通”工程；以强化社会治安综合治理、增强应急救援能力为重点的“平安”工程等五大工程。

促进群众增收。把增收富民作为政府托起民生的重要内容，加大因病致贫、返贫困难家庭的救助保障力度，帮助困难群众稳步脱困；推进农民工技能培训和就业环境改善，拓宽农民增收渠道，增加农民收入；提高劳动报酬在初次分配中的比重，建立企业职工工资正常增长机制，增加职工工资性收入；努力将公务员津贴补贴和事业单位绩效工资依政策落实到位；拓宽投资理财渠道，着力增加中等收入家庭的财产性收入，努力实现人民群众增收与国民经济增长的基本同步。

发展社会事业。稳步提高教育投入，改善农村办学条件，优化城区学校网点布局，完善八里湖新校区、科技中专新校区配套设施，促进各类教育均衡发展。加强教师队伍建设，深化教育教学改革，深入推进素质教育，提升基础教育发展水平。深化医药卫生体制改革，加强基层卫生服务能力建设，提高公共卫生应急能力，大力扶持中医药发展。加大文化遗产保护力度，加强文化市场管理，扎实推进县级图书馆、文化馆、档案馆和乡镇文化站建设。加快基层体育设施建设，广泛开展全民健身运动，提高竞技体育水平。纵深推进市场整治活动，完善食品药品监管体系，建设市食品药品检验中心，让人民群众吃上放心食品、用上放心药。加大市场监管力度，实施“阳光价格”工程，努力保持物价基本稳定。深化户籍制度改革，引导在城镇稳定就业和居住人员有序转为城镇居民。提高对气象、水文、地质等自然灾害的监测预警和防御能力。加强人口和计划生育工作，促进人口长期均衡发展。积极发展老龄事业，重视保护妇女儿童权益，加强残疾人康复机构建设。加快推进第二轮市志编纂工作。做好民族和宗教工作。深入开展国防教育，切实做好国防动员、民兵预备役、征兵和人民防空工作，落实优抚安置政策，开创“双拥”新局面。

维护和谐稳定。严格落实安全生产责任制和事故责任追究制，积极开展安全生产专项整治，有效防控各类安全事故发生。完善重大事项风险评估和突发公共事件应急管理机制。加强城乡社区管理，健全信访信息网络，排查、调处和化解各种矛盾纠纷。启动“六五”普法，加强法律援助工作。强化社会治安综合治理，依法防范和严厉打击各类犯罪，切实维护社会和谐稳定。

（七）加强政府自身建设，确保全面完成各项目标任务

始终保持奋发有为的精神状态，努力迈出争得九江应有地位的新步伐。做好2011年的各项工作，对于“十二五”的稳健开局，对于“三年翻番”的决战决胜，对于“五年进位”的顺利实现，意义重大，责任重大。我们将在市委的坚强领导下，以争得九江应有地位为己任，主动担当，履职尽责，不辱使命，不负重托；以更加饱满的热情、更加昂扬的斗志，把思想和精力全部集中到工作和事业上来，不懈怠，不分神，不松劲，凝成赶超发展的强大合力；以一往无前、连续作战的精神，一鼓作气，乘势而上，奋勇争先，全力夺取赶超发展的新胜利。

始终牢记执政为民的根本宗旨，努力迈出人民过上小康生活的新步伐。坚持“以人为本、执政为民”的理念，以实现人民过上小康生活为己任，牢固树立科学民生观，把保障和改善民生作为一切工作的出发点和落脚点，把群众满意不满意、高兴不高兴、答应不答应作为衡量政府工作的根本标准，作决策充分尊重民意，上项目充分珍惜民力，办实事充分体现民需，施改革充分保障民利，新增财力更多地向民生倾斜，努力解决人民群众最关心、最直接、最现实的问题，切实做到情为民所系、权为民所用、利为民所谋。继续提升政府“民声直通车”服务水平，拓宽服务领域，畅通服务渠道，着力打造群众“看得见，叫得应，用得上”的为民政府。

始终坚持依法行政的施政理念，努力迈出加强法治政府建设的新步伐。把依法行政贯穿于政府工作的全过程，切实增强法治意识，依法依规施政理政。自觉接受人大的法律监督和政协的民主监督，认真执行市人大及其常委会的决议决定，充分发挥市政协的政治协商和参政议政作用，广泛加强与各民主党派、工商联、人民团体和无党派人士的联系。进一步拓展政务公开的广度和深度，进一步健全科学民主的决策机制。继续深化行政审批制度改革，推进电子政务建设。加强执法监察和效能监察，做到令行禁止，确保政令畅通。

始终保持务实高效的工作作风，努力迈出加强机关效能建设的新步伐。深入开展“发展提升年”活动，围绕服务中心、服务基层、服务群众，进一步精简审批事项，优化办事流程，提高服务效率。坚持实事求是的思想路线和工作作风，深入一线，扑下身子，全力破解项目融资、用地、用工以及征地拆迁等重点领域的难题。充分调动班子成员和政府部门的积极性和创造性，在“三线工作法”的基础上，健全“六张作业单”推进机制，完善战线办公会、专题协调会、现场推进会等制度，畅通重大项目“绿色通道”，确保项目高效推进，工作快速落实，发展科学实现。

始终坚守清正廉洁的从政准则，努力迈出加强政府廉政建设的新步伐。严格执行党风廉政建设责任制，建立健全以优化发展环境、提高行政审批效率、强化国有资产监管、规范公共资源交易、确保财政资金阳光运行等为主要内容的惩治和预防腐败体系。深化工程建设领域突出问题专项治理，严肃查处规避招标、虚假招标、围标串标、违法分包等问题，严肃查办土地矿产资源审批、项目实施、工程质量、资金使用、产权交易、政府采购中的违纪违法案件。继续开展民主评议政风行风活动，坚决纠正损害群众利益的不正之风。加强财政审计、公共工程审计和领导干部经济责任审计。坚持厉行节约，严格控制一般性支出，勤俭办一切事业。严格遵守《廉政准则》8个严禁52个不准，不违反规定插手干预工程建设领域，不搞劳民伤财的“形象工程”和沽名钓誉的“政绩工程”，不利用职权和职务上的影响谋取不正当利益，努力打造一支政治坚定、业务精通、作风优良、廉洁高效的公务员队伍。

各位代表！赶超进位，目标所指；小康愿景，民心所向。让我们在省委、省政府和市委的坚强领导下，立足新起点，开启新征程，努力实现“十二五”的良好开局，为九江争得应有地位、人民过上小康生活而努力奋斗！

关联导读：

附件：
